# Nutritional factors and cross-national postpartum depression prevalence: an updated meta-analysis and meta-regression of 412 studies from 46 countries

**DOI:** 10.3389/fpsyt.2023.1193490

**Published:** 2023-06-15

**Authors:** Adi Fish-Williamson, Jennifer Hahn-Holbrook

**Affiliations:** Department of Psychology, University of California, Merced, Merced, CA, United States

**Keywords:** postpartum depression (PPD), diet, sugar sweetened beverages (SSBs), added sugar, global, national, review—systematic, meta analyses

## Abstract

**Background:**

Postpartum depression (PPD) is the most common complication associated with childbirth and can lead to adverse outcomes for both mothers and their children. A previous meta-analysis found that PPD prevalence varies widely across countries. One potential underexplored contributor to this cross-national variation in PPD is diet, which contributes to mental health and varies significantly around the world. Here, we sought to update the global and national estimates of PPD prevalence using systematic review and meta-analysis. Further, we examined whether cross-national variation in PPD prevalence is associated with cross-national variation in diet using meta-regression.

**Methods:**

To estimate national rates of PPD prevalence, we conducted an updated systematic review of all papers reporting PPD prevalence using the Edinburgh Postnatal Depression Scale between 2016–2021 and combined our findings with a previous meta-analysis of articles published between 1985–2015. PPD prevalence and methods were extracted from each study. Random effects meta-analysis was used to estimate global and national PPD prevalence. To examine dietary predictors, we extracted data on sugar-sweetened beverage, fruit, vegetable, total fiber, yogurt, and seafood consumption from the Global Dietary Database. Random effects meta-regression was used to test whether between-country and within-country variation in dietary factors predicted variation in PPD prevalence, controlling for economic and methodological variables.

**Results:**

412 studies of 792,055 women from 46 countries were identified. The global pooled prevalence of PPD was 19.18% (95% confidence interval: 18.02 to 20.34%), ranging from 3% in Singapore to 44% in South Africa. Countries that consumed more sugar-sweetened beverages (SSBs) had higher rates of PPD (Coef. = 0.325, *p* = 0.044, CI:0.010–0.680); Moreover, in years when higher rates of sugar-sweetened beverages were consumed in a country, there were correspondingly higher rates of PPD in that country (Coef. = 0.129, *p* = 0.026, CI: 0.016–0.242).

**Conclusion:**

The global prevalence of PPD is greater than previous calculations, and drastically varies by country. Sugar-sweetened beverage consumption explained some of the national variation in PPD prevalence.

## 1. Introduction

Mental illness is a leading cause of death and a major public health concern for countries around the world (1). Mental illness in mothers is particularly concerning, as it is not only a leading cause of maternal death in several countries around the world, but it can interfere with parenting behavior and lead to a multitude of adverse physical and emotional child developmental outcomes (2–4). Children with depressed mothers, for example, are more likely to experience psychopathology, behavior issues, and have lower academic achievement (5–7). The adverse impact of maternal depression on offspring may persist into adulthood, raising lifelong risk for mental health, behavioral, and relational issues throughout the lifespan (8–10). In this way, mental health issues can pass down through generations, highlighting the need to understand and prevent maternal mental illness.

Although awareness and research into maternal mental health has increased dramatically in recent decades, rates of mental illness in mothers is still high (11, 12). The most common mental health disorder in mothers is postpartum depression (PPD), defined by the DSM-5 as depressive symptoms experienced in the first 6 months postpartum (13). A recent meta-analysis of 291 studies from 56 countries estimated the global postpartum depression rate to be 17.7%, although prevalence rates varied dramatically by county (11). The lowest rates of PPD were found in Singapore (3%), Nepal (7%), and the Netherlands (8%); while the highest rates were found in Hong Kong (30%), South Africa (37%), and Chile (38%) (11). Examining country-level factors that might explain why rates of PPD vary so dramatically between nations could provide insight into the etiology of PPD and help inform government-led prevention programs. In an initial attempt to do this, Hahn-Holbrook and colleagues (11) conducted a meta-regression that revealed that cross-national variation in PPD prevalence was explained, in part, by economic disparities, fertility rates, and women's access to quality health care resources. Differences in research methodology used across countries explained very little of the cross-national variation in PPD prevalence. Notably, significant cross-national variation in PPD prevalence remained unexplained, suggesting the need to explore other cultural variables (11, 14).

One potential underexplored contributor to cross-national variation in PPD is diet, as diets vary significantly around the world and certain foods such as vegetables, fruit, legumes, nuts, dairy products, fish, and olive oil, may be protective against depression (15). Indeed, scholars have posited that the high levels of PPD seen in the modern day may be due, in part, to evolutionary “mismatches” between our diets today and the diets that humans ate throughout most of human evolution (16). Some of these changes include a decrease in fiber, omega-3 fatty acids, and micronutrients, and an increase in added sugar (16). Thus, we sought to examine whether national variation in dietary patterns predict cross-national variation in postpartum depression. Several dietary factors that have been previously linked to depression risk were considered including yogurt, fiber, fruit, vegetables, omega-3 fatty acids, and sugar-sweetened beverages (SSBs).

### 1.1. Added sugars

High added sugar consumption can adversely affect physical health and lead to obesity, type II diabetes, cardiovascular disease, and mortality (17–19). Several studies have reported links between added sugar consumption and depression (20, 21). However, very few studies have sought to examine the link between sugar consumption and PPD specifically, although one study from Taiwan found that SSB consumption was associated with higher scores on the Edinburgh postpartum depression scale (EPDS) (22).

Several mechanisms have been proposed as to how sugar consumption may increase depression risk. One proposed mechanism is that sugar consumption causes an increase in certain gut bacteria that disrupt healthy brain function, specifically memory (23). A previous study found that issues in working memory may underlie problems with regulating emotions that lead to mood disorders such as depression (23). Another possible mechanism discussed in previous research is the hypothalamic-pituitary-adrenal (HPA) axis. For example, Harrell and colleagues (24) found that diets high in fructose have the potential to alter HPA function in rats, which was associated with increased risk for depressive-like symptoms (24). As fructose is the most common sweetener in processed foods, especially in SSBs, it is plausible that similar effects could be seen in humans (25). Given that SSBs are the main source of added sugar in diets around the globe (26–29), we sought to test whether countries that consume more SSBs have high higher rates of PPD.

### 1.2. Seafood

Several studies have also linked higher seafood consumption to reduced risk of depression, and postpartum depression specifically. For example, in a systematic review of six studies, Opie and colleagues (30) concluded that seafood consumption was protective against PPD (30). Another study by Hibbeln (31) examined this relationship at the national level and found that countries that consumed more seafood had lower rates of PPD (31), although national rates of PPD in this study were not derived by meta-analysis, calling into question their relatability. Authors of these studies have suggested that seafood may reduce PPD risk because it is high in omega-3 fatty acids, which can be depleted in mothers during pregnancy.

There are several mechanisms that have been theorized to explain how seafood consumption may reduce depressive symptoms and most focus on the role of omega-3 fatty acids. One important type of such acids is DHA, which is abundant in the brain and alters functions of neural systems that utilize dopamine and serotonin, which play a role in depression (32). Another explanation is that omega-3 fatty acids have anti-inflammatory properties and can reduce the production of pro-inflammatory cytokines that contribute to depressive symptoms (33). In addition, emerging research suggest that omega-3 fatty acids benefit the gut microbiome (34), which is also critical for mental health. Given the previously established connection between seafood consumption and depression, we aimed to explore if national variation in seafood consumption predicted national variation in PPD prevalence using meta-analytically derived national PPD prevalence estimates.

### 1.3. Yogurt and Probiotics

Another dietary factor that varies by country is probiotic consumption, which introduces live microorganisms to the gut, altering the microbiome, which may have benefits for the prevention and treatment of depression (35). One of the pathways through which probiotic consumption is thought to reduce the risk of depression is through the gut-brain axis, the bidirectional system that directly and indirectly allows for microbes in the gut to communicate with the brain (36). Through this axis, microbes can affect the brain by regulating HPA functions, and reducing associated pro-inflammatory cytokines, both of which are heavily implicated in the etiology of depression (36). A recent systematic review concluded that probiotic consumption is a promising avenue for the prevention and treatment of depression, although the review did not focus on PPD specifically (37).

Given that there is significant overlap between major depression and PPD, several studies have examined the effectiveness of probiotic consumption for PPD prevention. For example, a randomized controlled trial found that women who received probiotic supplements during pregnancy (*N* = 193) reported significantly fewer symptoms of postpartum depression than women in the placebo group (*N* = 187) (38). These results are consistent with rodent models, in which administering probiotics improves depression-like symptoms in highly stressed rat mothers by altering gut microbiota composition, brain monoamines, oxidative stress, and reversing stress-induced changes in the HPA-axis and brain-derived neurotrophic factor (BDNF) (39). Probiotic supplementation is an effective way of distributing and controlling probiotics exposure in experimental research, however, to examine the effects of probiotics on PPD from a global perspective, focusing on traditional foods containing probiotics may be more fitting.

Although there are many sources of probiotics in diets across the world, yogurt is one of the most ancient and most popular (40, 41). References to yogurt consumption for its health benefits date back to 6000 BC in Indian Ayurveda scripts, although it was not until the 1900s that these benefits were attributed to probiotics (40). Patterns of yogurt consumption vary greatly by country. For example, the majority of people in France consume yogurt every day, which is the case for only 6% of Americans. Research conducted in 15 countries showed that the largest amounts of yogurt were consumed in the Netherlands, France, Turkey, Spain, and Germany. The smallest amounts were consumed in Egypt, Colombia, Russia, Romania, and South Africa (42). In a cohort of 14,539 participants, high consumption of whole-fat yogurt was associated with lower rates of depression in women compared to those who did not consume as much yogurt (43). An additional study of 9,965 participants found that yogurt consumption had a protective effect on depression (44). Thus, it seems plausible that cross-national variation in yogurt consumption might explain some of the national variation in PPD prevalence.

### 1.4. Fiber

Fiber consumption also varies significantly by country, for example, Brazil's average fiber consumption (12.3 grams/day) is approximately half of Sweden's (23–25 grams/day) (45). Moreover, high fiber consumption has been found to reduce the risk of depressive symptoms (46, 47). One theory to explain this relationship is through the effect of fiber on the gut microbiota (46). Gut microbes can break down foods the human digestive system alone is not easily able to digest (e.g., insoluble fiber found in whole grains, nuts, and certain fruits and vegetables), creating a mutually beneficial relationship (48). Non-human animal studies suggest that fiber consumption may lead to microbiota-driven modification of gene expression and increased production of neurotransmitters that may protect against depressive symptomatology (47). In addition to its benefits for the microbiome, fiber also has anti-inflammatory properties thought to help reduce depression risk (47). Specifically, consuming a diet high in fiber may lower inflammation by modifying the pH and permeability of the gut, and reducing inflammatory compounds that can alter neurotransmitter concentrations and influence depressive symptoms (47).

To the authors' knowledge, no human studies have examined the relationship between insoluble (or any) fiber consumption and postpartum depression risk. However, one rodent study found that high intake of dietary fiber supplements alleviates depression-like symptoms postpartum in female mice (49). Therefore, it seems possible that dietary fiber consumption could play a role in PPD such that countries that consume less fiber have higher rates of PPD.

### 1.5. Fruits and vegetables

Several studies have found that mothers who consume diets that include plenty of fruits and vegetables have a lower risk of developing PPD (15, 30, 50). Fruits and vegetables contain high levels of fiber, and so it is perhaps not surprising that diets rich in fruits and vegetables are both associated with decreased inflammation, which is a known risk factor for PPD (51, 52). Additionally, as fiber is the key food source for may beneficial gut-bacteria, adequate fruit and vegetable intake is associated with higher bacterial diversity in the gut microbiome (53), which may be protective against PPD (53). In line with the view that fruit and vegetable consumption may be protective against PPD, a cross-sectional survey of 939 women found that higher consumption of fruits and vegetables was associated with lower likelihood of reporting PPD symptoms (54). Thus, it seems feasible that countries that consume more fruits and vegetables may have lower rates of PPD than countries that consume fewer fruits and vegetables.

### 1.6. The current study

The aims of the current study were four-fold. First, we sought to update the global and national estimates of PPD prevalence provided by Hahn-Holbrook et al. (11) by conducting a systematic review and meta-analysis of studies published between 2016 and 2021. The second aim was to conduct a meta-regression to examine whether the dramatic differences in PPD prevalence by country can be explained by cultural variation in dietary factors like sugar, yogurt, fiber, fruit and vegetable, and seafood consumption. We hypothesized that countries with higher yogurt, fiber, fruit, vegetable, and seafood consumption, on average, would report lower levels of PPD. Moreover, we predicted that countries with higher SSB consumption would report higher levels of PPD. Given that diets in a country can and do change overtime, the third aim of this study was to explore if changes in a country's diet over time corresponded to changes in prevalence rates of PPD over time (hereafter termed within-country variation in PPD). We predicted that, in years when countries consumed more sugar and less yogurt, fiber, fruits, vegetables, and seafood, studies within those countries would report higher levels of PPD. Finally, a fourth study aim was to explore the extent to which methodological differences across studies contributed to variation in PPD prevalence across studies and across countries.

## 2. Methods

### 2.1. Research design

This study was carried out in a six-step process. First, we conducted a systematic review of studies reporting PPD prevalence published since the meta-analysis published in 2018 by Hahn-Holbrook and colleagues. Following PRISMA guidelines (55), we extracted information on PPD prevalence and methodological variables from each article. Second, this new dataset was merged with Hahn-Holbrook and colleagues (11) dataset, giving us a database of studies reporting PPD prevalence using the EPDS scale published between 1985 and 2021. Third, we used meta-analysis to estimate an updated global rate of PPD prevalence, as well as updated PPD prevalence estimates by country. Fourth, we conducted a meta-regression to explore the extent to which methodological variation predicted variation in PPD prevalence reported across studies. Fifth, using our meta-analytically derived PPD prevalence estimates for each country, we used meta-regression to test the extent to which national variation in rates of PPD could be explained by cultural variation in dietary factors. In these analyses, we statistically controlled for methodological conventions used across countries and national GDP, as the previous meta-analysis by Hahn-Holbrook et al. (11) identified national poverty as the strongest predictor of cross-national variation PPD. Finally, we conducted a meta-regression to explore whether within-country (mean-centered) variation in dietary consumption patterns across time predicted within-country (mean-centered) variation in rates of PPD across time.

### 2.2. Search strategy and study selection criteria

In order to update the global and national estimates of PPD prevalence provided by Hahn-Holbrook et al. (11), who included articles published between January 1, 1985 to December 31st 2015, we followed the same search and methodological strategies. To identify new potentially eligible articles published between January 1, 2016 and July 26, 2021, we searched PubMed, PsychINFO, and CINAHL using a combination of the following MeSH terms in the abstract: (“postpartum depression” or “postnatal depression”) and (“incidence” or “prevalence”). Additionally, we used the measures and instruments qualifier “Edinburgh Postnatal Depression Scale”. The Edinburgh Postpartum Depression Scale (EPDS) is a 10-item self-report, widely-used tool specially designed to measure PPD. We chose to focus on studies that used the EPDS specifically, as this is overwhelmingly the most commonly used tool to measure postpartum depression; 70% of studies that measure PPD use the EPDS (11). Moreover, this measure has been specially designed and validated to detect depressive symptoms in the postpartum period. Other self-report depression scales can be problematic to use in the postpartum period as they contain items about weight gain/loss and sleep changes, which are normal in the postpartum period. Clinical interviews for depressive status typically produce lower prevalence rates than self-report measures. However, we chose to focus on studies reporting prevalence using the gold-standard self-report measure (rather than clinical interviews) given that clinical interviews are rarely conducted in low-income countries given the increased cost and participant burden. We further narrowed our search by only including studies of human females published in English. The exact Boolean searches used for each database are provided in Supplementary material.

To be included in this meta-analysis, studies were required to report PPD prevalence using the EPDS on samples of mothers ≤ 1 year postpartum with a sample size over 20 (56). To address the important issue of the timing of depression postpartum, we coded studies in terms of when in the postpartum period that depression was assessed, allowing us to examine this variable using meta-regression. We also excluded studies reporting PPD prevalence in samples unlikely to be representative of the general population (e.g., studies that exclusively recruited special populations like women with a history of depression, adolescent mothers, mothers seeking treatment for depression, mothers of high-risk infants, etc.).

**Figure 1:**
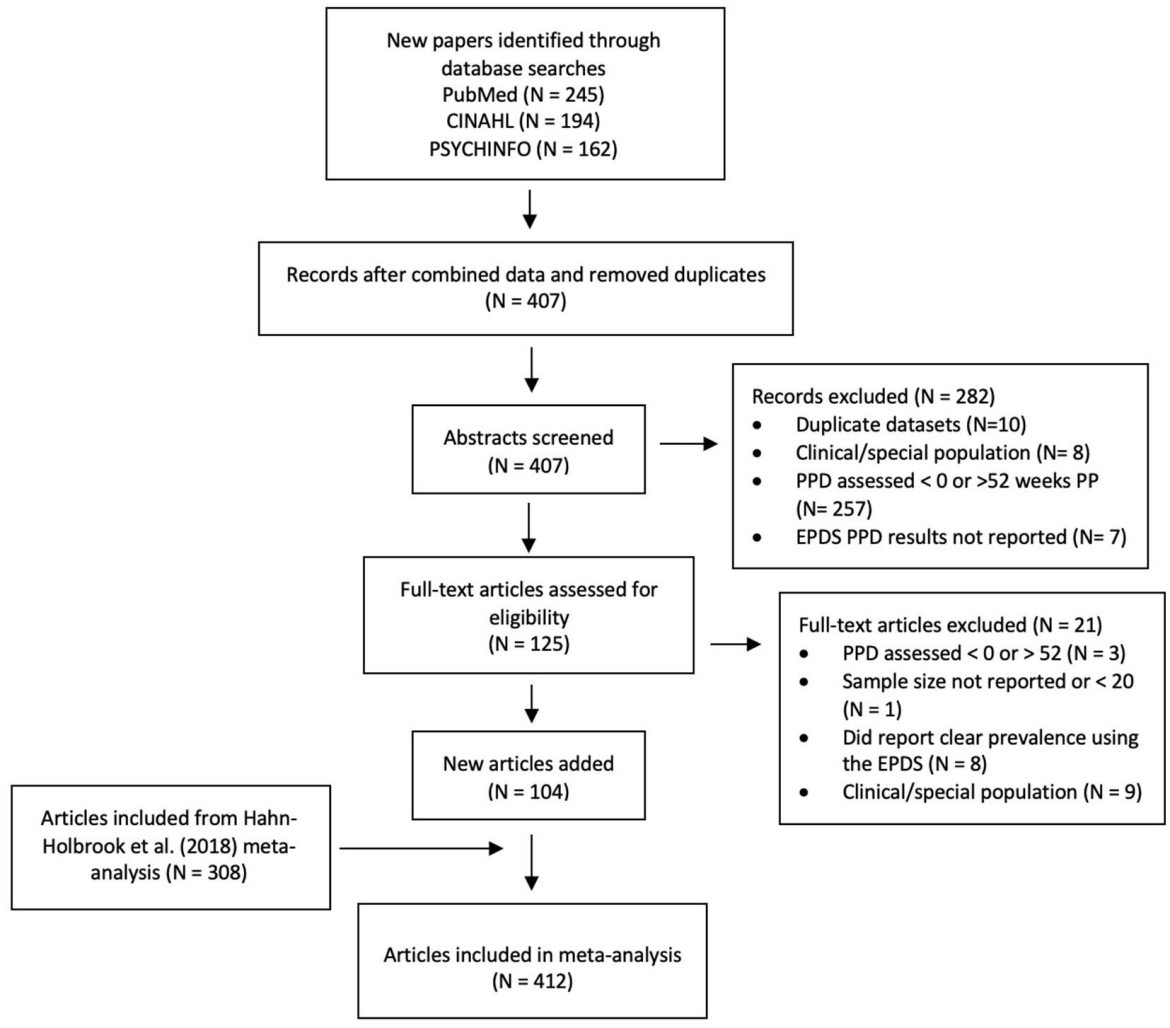
PRISMA flow diagram reporting identification and selection of studies for the meta-analysis.

See Figure 1 for a PRISMA flow diagram reporting identification and selection of studies for this updated meta-analysis. Of the 601 studies that our updated search produced, 407 abstracts were reviewed, and 125 full text articles were assessed for eligibility. Of these, 104 studies published between January 2016 and July 2021 met our inclusion criteria. These studies were coded for methodological and PPD prevalence, and then this dataset was merged with the from Hahn-Holbrook et al. (11), resulting in a full dataset of 412 studies that could be included in this updated meta-analysis.

### 2.3. Data extraction

The following methodological variables were coded from each study: PPD prevalence, total sample size, EPDS cutoff score employed, and the timeframe postpartum in which PPD was assessed. To get one estimate of PPD prevalence per study, data from longitudinal studies reporting PPD in the same women at multiple time points were merged by averaging the PPD prevalence over the time points weighted by the sample size at each time point. Also, if multiple prevalence rates were reported in the same study using different EPDS cutoffs, the prevalence rate from the lowest EPDS cutoff was chosen by default. This decision could cause a bias toward higher estimates of PPD incidence; therefore, we also used meta- regression to estimate PPD prevalence at the standard recommended EPDS cutoffs for possible (9/10) and probable (12/13) PPD (56). To investigate whether studies including women earlier or later in the postpartum period report higher PPD prevalence, we coded the range of the timeframes postpartum during which PPD was assessed and used this score to predict PPD prevalence in meta-regression.

### 2.4. Methodological variables

Previous studies indicated that some of the differences in PPD prevalence found across studies and between countries can be explained, in part, by methodological differences (11, 57). For example, it is possible that scientists in some countries use lower PPD cutoffs scores more often than scientists in other countries, leading to the appearance of cross-national variation in PPD, when really it is just that some countries set lower symptom thresholds than others. To investigate the role of methodological differences in cross-national PPD prevalence, national averages for each study N, EPDS cutoff score, age of baby at start of data collection, length of assessment window, and year published were included in meta-regression models.

### 2.5. Dietary factors

This study examined several dietary factors that have been associated with depression in previous research, namely: fiber, yogurt, seafood, SSBs, vegetables, and fruit. To assess national rates of dietary consumption, we used estimates collected by the Global Dietary Database (GDD) (58). The database is part of the Global Nutrition and Policy Consortium, an initiative based at the Tufts Friedman School of Nutrition Science and Policy. GDD data was collected through nationally representative surveys when available, and, when national surveys were not available, multiple smaller surveys that encompassed people from different parts of the country were aggregated. For countries with no surveys available, sources such as the WHO Infobase were used (58). We used the national consumption of foods measured in terms of grams per day, excluding dietary supplements (e.g., pills). Dietary fiber includes intake from all food sources, including fruits, vegetables, grains, legumes, and pulses. Yogurt data includes total intake of all types of yogurts and fermented milk. Seafood includes the total daily intake of fish and shellfish. SSB data includes intake of any beverage with added sugars having ≥ 50 kcal per 8 oz or 236.5 g of added sugar per serving, including commercial or homemade beverages, soft drinks, energy drinks, fruit drinks, punch, lemonade, and frescas. The SSB variable excludes fruit and vegetable juices with no added sugar, and non-caloric artificially sweetened beverages. Fruit includes total intake of fresh, frozen, cooked, canned, or dried fruit, and excludes fruit juices and salted or pickled fruits. Vegetables include total intake of fresh, frozen, cooked, canned, or dried vegetables, and excludes vegetables juices, salted or pickled vegetables, starchy vegetables such as potatoes, and legumes such as beans. Given that dietary patterns in a country can and do change over time, food consumption in the GDD is reported in yearly intervals. This within-country variation in food and beverage consumption was taken into account in our analysis by mean-centering food consumption within each country, giving us a variable that captures whether consumption of each food was higher or lower in a given year than the country's national average.

### 2.6. National GDP

Previous research has identified Global Domestic Product (GDP) per capita as a significant predictor of cross-national PPD prevalence (11). As GDP may relate to national dietary habits, we wanted to statistically adjust for this factor in models testing the association between PPD and dietary factors. National GDP data (in adjusted US dollars) were obtained from the World Bank (59).

### 2.7. Data analytic strategy

Following recommendations for meta-analysis of prevalence (60), we used a double-arcsine transformation of the PPD prevalence data before calculating the study weights and 95% confidence intervals (CIs) to avoid undue influence of weights obtained for studies with low or high prevalence (prevalence close to 0 or 1). To test for heterogeneity in the data, both the Cochran *Q* test statistic and the *I*^2^ statistic were conducted (61). The same procedure was followed to create meta-analytically derived national estimates of PPD prevalence based solely on the studies available from each country. Meta-analytic estimates of PPD prevalence could not be calculated in countries with fewer than two studies (*N* = 21) (62). All meta-analyses were conducted using the program MetaXL and the “prev” command (60).

Three sets of meta-regressions were performed, the first addressing which methodological factors predicted variation in PPD across all studies, regardless of the nation in which the study was conducted. The second addressed methodological and dietary predictors of PPD variation across nations. The third addressed methodological and dietary predictors of PPD variation within-nations across time. All meta-regression analyses were performed in STATA 14 (63) using the “metareg” command with random-effects models (because all tests indicated significant heterogeneity). To obtain the standard errors needed to weight studies (or nations) for meta-regression in STATA, we transformed the 95%-CIs provided by MetaXL using the following formula (upper 95% CI—lower 95% CI)/3.92.

Funnel plots were used to test whether papers were more or less likely to be published if they had higher or lower PPD prevalence. Results were considered statistically significant if *p*-values were under 0.05. Effect sizes are reported as unstandardized coefficients and *R*^2^ values obtained in meta-regression models.

## 3. Results

### 3.1. Meta-analysis of global PPD prevalence

**Figure 2:**
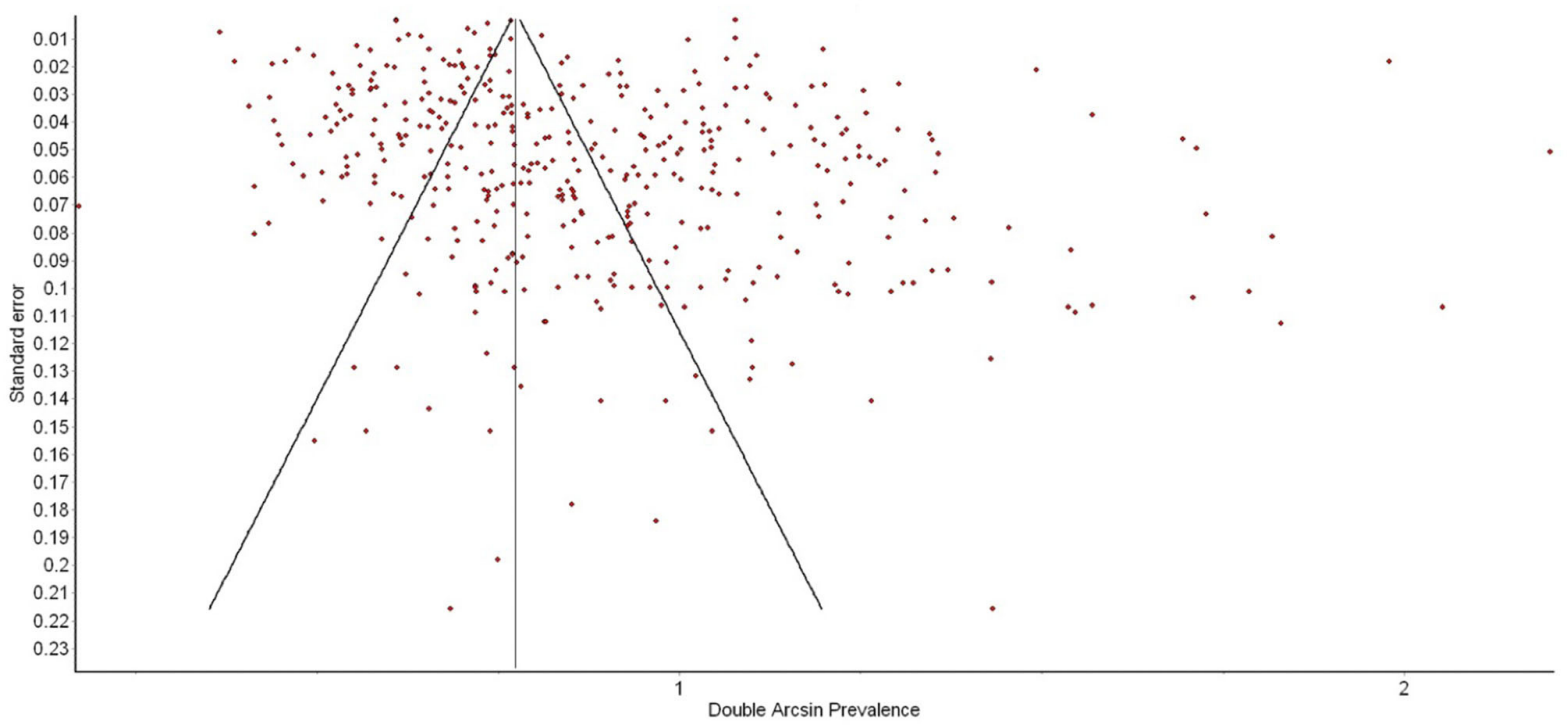
Funnel plot of postpartum depression (PPD) prevalence as a function of prevalence estimate standard error. There was no evidence of publication bias.

792,055 women from 412 studies were included in this meta-analysis. Table 1 presents the data extracted from the 103 new studies published since the Hahn-Holbrook and colleagues (11) meta-analysis. The updated global pooled prevalence of PPD across all 412 studies was 19.18% (95% CI: 18.02 to 20.34%). There was a significant degree of heterogeneity between studies (*Q* = 40,688.45, *p* = 0.00, *I*^2^ = 99%). Adjusting for the recommended EPDS cut-offs yielded a global PPD prevalence of 19.9% (CI: 18.31 to 21.49%) for possible PPD (EPDS cutoff of 10) and 18.28% (CI: 16.56 to 19.9%) for probable PPD (EPDS cutoff of 13). There was no evidence of publication bias as a function of PPD prevalence rate reported in studies (see Figure 2).

**Table 1:** New studies included in this meta-analysis published January 2016–July 2021.

**Reference**	***n***	**Depression prevalence (%)**	**Cut-off used**	**Postpartum assessment (weeks)**	**Country**
Ogbo et al. (64)	17,564	0.03	13	0–6	Australia
Azad et al. (65)	376	0.39	10	0–52	Bangladesh
Abuchaim et al. (66)	205	0.31	10	0–9	Brazil
Avilla et al. (67)	287	0.13	11	4–6	
Corrêa et al. (68)	3,060	0.20	11	4–14	
Farías-Antúnez et al. (69)	3,838	0.28	10	52	
Halal et al. (70)	2,222	0.28	13	13	
Lei et al. et al. (27)	10,223	0.26	13	26–52	
Lorentz et al. (71)	50	0.35	10	0–26	
Dennis et al. (72)	97	0.08	10	1–8	Canada
Emerson et al. (73)	46,863	0.13	10	6–12	
Falah-Hassani et al. (74)	522	0.24	10	1–8	
Gan et al. (75)	2546	0.11	10	6	China
Guo et al. (76)	438	0.22	9.5	6	
Huang et al. (77)	241	0.10	9.5	0–26	
Li et al. (78)	240	0.16	12	1–4	
Liang et al. (79)	864	0.30	10	6–12	
Liu et al. (80)	1,204	0.23	13	6	
Liu et al. (81)	882	0.07	11	0–4	
Long et al. (82)	242	0.13	13	26	
Peng et al. (83)	1,325	0.27	10	2–8	
Peng et al. (84)	4,813	0.12	10	6	
Shi et al. (85)	213	0.16	12	0	
Wang et al. (86)	1,126	0.12	13	2–8	
Xiong et al. (87)	468	0.56	10	6	
Zhou et al. (88)	288	0.10	9	0–8	
Zhou et al. (89)	228	0.26	13	4	
Ding et al. (90)	308	0.12	10	6	
Stylianides et al. (91)	543	0.28	12	6	Cyprus
Fiala et al. (92)	3,233	0.11	13	6–26	Czechia
Ahmed et al. (93)	257	0.34	13	9–26	Egypt
Meky et al. (94)	170	0.04	14	8–16	
Adamu et al. (95)	618	0.23	13	0–6	Ethiopia
Dadi et al. (96)	916	0.09	6	2–8	
Dadi et al. (97)	866	0.09	13	26	
Wubetu et al. (98)	308	0.16	13	0–6	
Fritel et al. (99)	1,413	0.13	10	16–35	France
Koutra et al. (100)	1,037	0.14	13	8	Greece
Ana et al. (101)	1,406	0.32	13	0	India
Badiya et al. (102)	347	0.09	10	0–13	
Jha et al. (103)	1,004	0.17	10	0	
Joshi et al. (104)	300	0.19	10	0–50	
Murry et al. (105)	284	0.22	12	6–8	
Nurbaeti et al. (106)	283	0.20	13	4–13	Indonesia
Nurbaeti et al. (107)	166	0.20	12	4	
Abdollahi et al. (108)	1,910	0.19	12	13	Iran
Ezzeddin et al. (109)	325	0.35	13	13–35	
Afshari et al. (110)	505	0.39	12	2–26	
Iranpour et al. (111)	360	0.35	13	0–13	
Daoud et al. (112)	1,128	0.10	10	6–26	Israel
Mazor et al. (113)	120	0.33	10	0	
Bruno et al. (114)	110	0.05	10	13–26	Italy
Clavenna et al. (115)	2,706	0.05	13	8–12	
Cozzolino et al. (116)	105	0.19	10	0–52	
Ostacoli et al. (117)	163	0.44	11	0–3	
Cui et al. (118)	80,872	0.09	9	4	Japan
Honjo et al. (119)	86,490	0.09	9	4	
Iwata et al. (120)	2,709	0.12	9	4–26	
Matsumura et al. (121)	90,194	0.13	9	4–26	
Okubo et al. (50)	1,316	0.08	9	8–40	
Muchanga et al. (122)	82,489	0.14	9	0–5	
Shibata et al. (123)	258	0.15	9	0	
Suzuki et al. (124)	809	0.14	9	4	
Takehara et al. (125)	1,306	0.12	9	0–12	
Zejnullahu et al. (126)	247	0.21	12	6	Kosovo
Inthaphatha et al. (127)	428	0.32	10	6–8	Lao People's Democratic Republic
Badr et al. (128)	150	0.19	13	0	Lebanon
Radzi et al. (129)	387	0.80	10	0–52	Malaysia
Abdul et al. (130)	458	0.20	13	4–13	Maldives
Suárez-Rico et al. (131)	293	0.39	13	4–12	Mexico
Bhusal et al. (132)	346	0.17	13	4–14	Nepal
Chalise et al. (133)	242	0.17	12	0–26	
Maharjan et al. (134)	330	0.15	13	0–12	
Sulyman et al. (135)	483	0.22	13	0	Nigeria
Shakeel et al. (136)	643	0.09	10	14	Norway
Qandil et al. (137)	97	0.33	11	1–25	Palestine
Labrague et al. (138)	165	0.16	10	6	Philippines
Drozdowicz-Jastrzebska et al. (139)	84	0.12	12	0	Poland
Jaeschke et al. (140)	434	0.15	13	6–12	
Maliszewska et al. (141)	546	0.33	8	0	
Nasr et al. (142)	174	0.39	13	0–24	Saudi Arabia
Almutairi et al. (143)	113	0.26	15	1–6	
Alzahrani et al. (144)	217	0.17	13	8–10	
Mokwena et al. (145)	406	0.57	13	0–52	South Africa
Fan et al. (146)	1,349	0.12	9	1–4	Sri Lanka
Khalifa et al. (147)	223	0.06	12	13–35	Sudan
Eckerdal et al. (148)	3,888	0.13	12	6	Sweden
Roumieh et al. (149)	1,105	0.28	13	4–7	Syria
Lin et al. (150)	474	0.16	13	26	Taiwan
Lin et al. (151)	344	0.08	10	6–8	
Lin et al. (152)	180	0.09	7	4	
Bay et al. (153)	550	0.25	13	0–4	Turkey
Bolak Boratav et al. (154)	87	0.48	12	13–26	
Çelik et al. (155)	63	0.43	13	8	
Demirel et al. (156)	461	0.31	13	2–52	
Dikmen-Yildiz et al. (157)	858	0.26	13	4–26	
Sahin et al. (158)	497	0.05	13	4–6	
Yilmaz et al. (159)	530	0.25	13	8–52	
Alhammadi et al. (160)	504	0.33	10	1–26	United Arab Emirates
Emerson et al. (73)	43	0.12	10	9–26	USA
Kothari et al. (161)	249	0.04	12	2–26	
Magliarditi et al. (162)	970	0.12	10	0	
Soffer et al. (163)	1,113	0.07	10	6	
Do et al. (164)	116	0.28	12	0–52	Vietnam

### 3.2. Meta-regression of between-study variation

Table 2 presents the results of the meta-regression testing the extent to which methodological variables predict variation in PPD prevalence across studies. We found that studies with smaller sample sizes tend to report higher levels of PPD prevalence (Coef. = −0.117, *p* = 0.019; CI: −0.215 to −0.019). Studies that used lower EPDS cutoff scores reported significantly higher PPD prevalence (Coef. = −0.105, *p* = 0.44; CI: −0.207 to −0.003). Studies with a longer window of PPD assessment tended to report higher levels of PPD (Coef. = 0.161, *p* = 0.003, 95% CI: 0.056 to 0.266). No other methodological variables predicted between-study variation in PPD. Together, methodological variables accounted for 4.63% of the variance in PPD prevalence between studies [*F*[5, 374] = 4.68, *p* < 0.05].

**Table 2:** Between study predictors of postpartum depression prevalence.

	**Coeff**.	**P-value**	**95% CI**	
Study *N*	−0.117	0.019	−0.215	−0.019
EPDS cutoff score	−0.105	0.044	−0.207	−0.003
Age of baby at start	−0.174	0.001	−0.277	−0.071
Assessment window	0.161	0.003	0.056	0.266
Year published	−0.022	0.664	−0.096	0.079

### 3.3. Meta-analyses of national PPD prevalence

**Figure 3:**
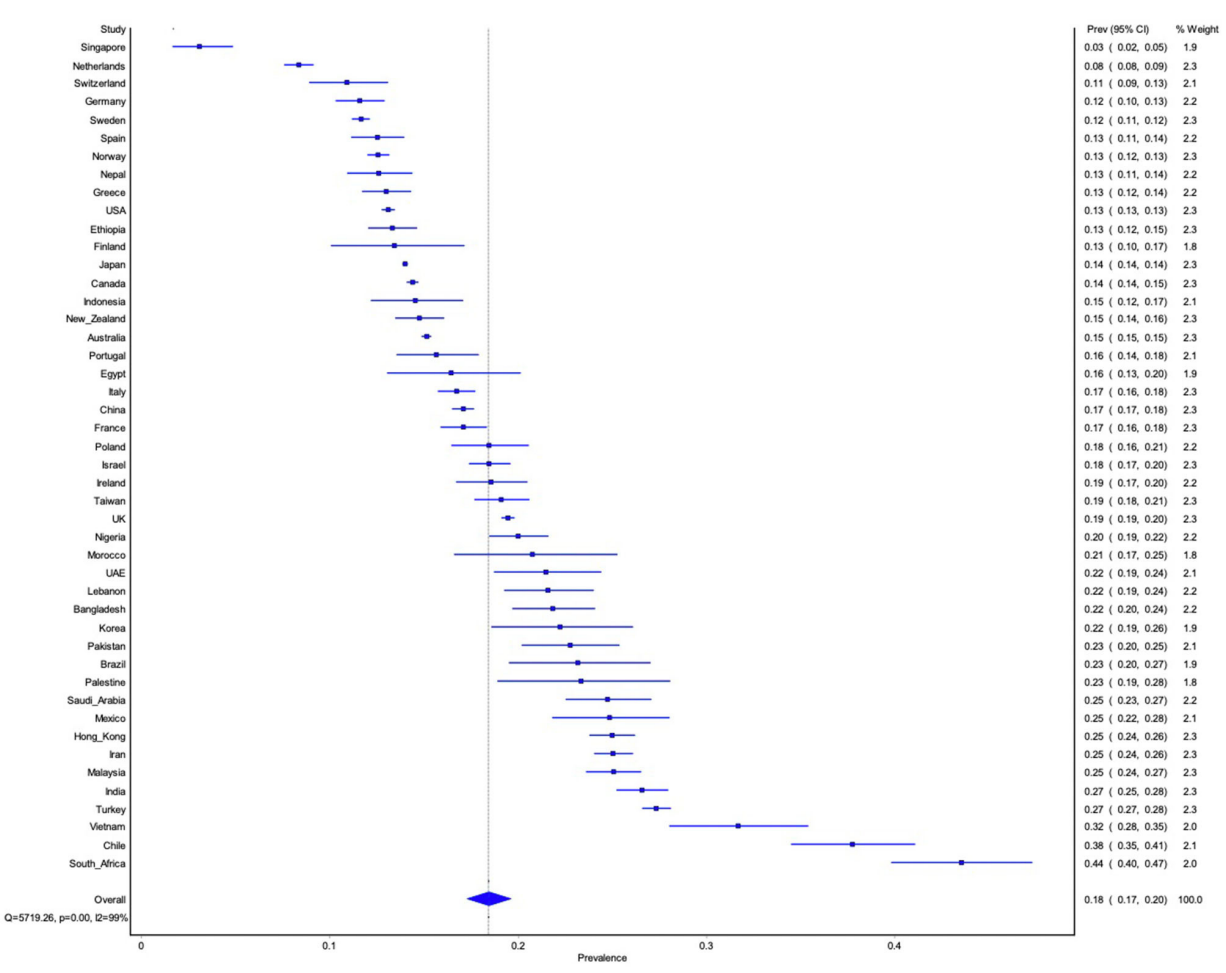
Forest plot of between-country variation in PPD prevalence.

See Figure 3 for meta-analytically derived estimates of PPD prevalence in 46 countries. National sample sizes ranged from 332 to 353,444 women. National estimates of PPD ranged from 3% in Singapore, 8% in Netherlands, and 11% in Switzerland, to 32% in Vietnam, 38% in Chile, and 44% in South Africa. Meta-analysis suggested that there was significant heterogeneity in PPD prevalence between nations (*Q* = 8,130.37 *p* < 0.00, *I*^2^ = 99%), suggesting the need for meta-regression to explain cross-national variation in PPD prevalence.

### 3.4. Meta-regression of predictors of between-country variation

**Figure 4:**
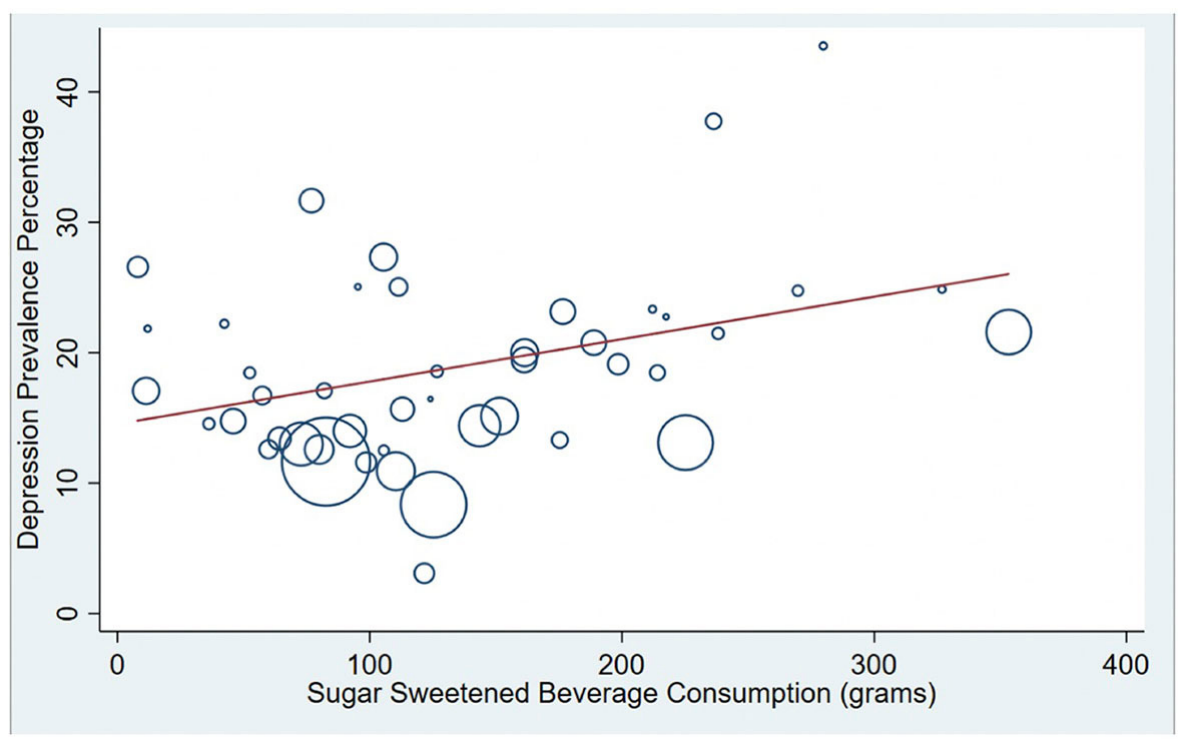
Correlation between National PPD prevalence and National Sugar Sweetened Beverage (SSB) consumption. Bubble plots are presented showing the significant positive association between SSB consumption and national postpartum depression (PPD) prevalence (R-squared = 11.4%). Countries with larger bubbles had larger sample sizes and were weighted accordingly in meta-regression models.

See Table 3 for the results of a meta-regression in which national averages of methodological variables, along with economic and dietary factors, were entered together to predict cross-national variation in PPD prevalence. Of the methodological variables, only the timing of the start of the assessment window predicted cross-national variation in PPD prevalence, with nations that tended to measure PPD earlier in the postpartum reporting higher PPD levels (Coef. = −0.413 *p* = 0.037; CI: −0.800 to −0.027). Methodological variables alone (when dietary and economic factors were removed from the model) accounted for 10.88% of the total between-country variation in PPD prevalence. GDP did not predict cross national PPD prevalence when methodological and dietary factors were included in the model. In terms of dietary factors, SSB consumption emerged as the only significant dietary factor that predicted cross-national PPD prevalence. This effect remained statistically significant when methodological, economic, and other dietary factors were included in the model. Specifically, countries that consumed more SSBs had higher levels of PPD (Coef. = 0.345, *p* = 0.044; CI:0.010 to 0.680). SSB consumption alone accounted for 11.42% of between-study variation in PPD prevalence (see Figure 4).

**Table 3:** Between-country predictors of postpartum depression prevalence.

	**Coeff**.	**P-value**	**95% CI**	
Study *N*	1.335	0.434	−2.121	4.790
Age of baby at start	−0.413	0.037	−0.800	−0.027
Assessment window	−0.144	0.589	−0.684	0.396
EPDS cutoff score	0.220	0.524	−0.480	0.919
GDP	−0.238	0.318	−0.719	0.243
Fiber consumption	0.016	0.935	−0.376	0.408
Yogurt consumption	−0.017	0.933	−0.434	0.400
Seafood consumption	−0.103	0.513	−0.425	0.218
SSB consumption	0.344	0.044	0.010	0.679
Vegetable consumption	0.064	0.747	−0.338	0.465
Fruit consumption	−0.160	0.416	−0.559	0.238

### 3.5. Meta regression of within-country variation

Given that this study includes research on PPD conducted over 40 years, it is important to consider the fact that dietary patterns within a country can change dramatically over time. For example, daily SSB consumption in Japan was approximately 109 grams per person, per day in 1997 but has decreased to about 86 grams in 2018, representing a 21.1% decrease over time. Therefore, we examined whether postpartum depression rates tended to be higher in a country in years when they ate more or less of certain foods. To do this, we matched the year that the PPD study was published to the dietary behavior in that country for that given year. Then, to isolate within-country variation, we created country specific z-scores that mean-center the dietary intake for each country over time, with higher scores representing more consumption of a dietary factor relative to that country's average consumption over-time. We then used these country mean-centered scores to predict country mean-centered variation in PPD (see Table 4 for results). We found that, in years when countries consumed more SSBs, studies within that country tended to report higher levels of PPD (Coef. = 0.129, *p* = 0.026; CI:0.016 to 0.245. In terms of methodological variables, when studies within a country measured PPD earlier and using a lower EPDS cutoff, compared to the county's average, the studies tended to report higher PPD.

**Table 4:** Within-country variation in dietary and methodological factors predicting within-country variation of postpartum depression prevalence across time.

	**Coeff**.	**P-value**	**95% CI**	
Study *N*	−0.165	0.002	−0.271	−0.059
EPDS cutoff score	−0.259	0.000	−0.366	−0.153
Age of baby at start	0.018	0.787	−0.112	0.148
Assessment window	0.126	0.023	0.017	0.234
Year of publication	−0.082	0.245	−0.219	0.056
Fiber consumption	−0.001	0.982	−0.125	0.122
Yogurt consumption	−0.034	0.650	−0.179	0.112
Seafood consumption	0.070	0.260	−0.052	0.193
SSB consumption	0.129	0.026	0.016	0.243
Vegetable consumption	−0.078	0.287	−0.223	0.066
Fruit consumption	−0.067	0.367	−0.214	0.079

All variables were Z scored and mean centered.

## 4. Discussion

This study represents the largest meta-analysis and meta-regression on postpartum depression prevalence in the literature to date. We found that the unadjusted global prevalence rate of PPD in studies utilizing the EPDS scale is 19.18% (95% CI: 18.02 to 20.34%). Adjusting for the recommended EPDS cutoffs yielded a prevalence of 19.9% for possible PPD (>10) and 18.28% for probable PPD (>13). These estimates are slightly higher than the previous calculation by Hahn-Holbrook et al. (11), which found a global PPD prevalence of 17.7% (95% CI: 16.6–18.8%) (11). One explanation for this discrepancy is that the current study adds new data with a higher proportion of studies from relatively low- and middle-income countries, that tend to report higher rates of PPD (57). Between South Africa, which has the highest PPD prevalence rate (44%), and Singapore, which has the lowest PPD prevalence rate, there is a 41% difference. Considering there are many low-income countries missing from this analysis, it is plausible that global PPD is even more prevalent than our findings suggest. While several methodological variables predicted cross-national variation in PPD, specifically the timing of assessment and EPDS cutoff used, the strongest individual predictor in this study was SSB consumption.

Our meta-regression revealed that SSB consumption predicted PPD prevalence both between-countries and within-countries. Specifically, countries that consumed more SSBs, on average, had higher rates of PPD prevalence. This finding alone could be attributed to other cultural differences that co-vary with SSB consumption, although this effect was still significant when methodological factors and GDP were included in the model. However, we also found that, within the same country, in years with higher SSB consumption, studies tended to report higher rates of PPD in that country. This within-country finding lends credence to the hypothesis that it is SSB consumption, and not some other cultural factor, that contributes to higher rates of PPD.

The current study did not find a statistically significant association between seafood consumption and cross-national PPD prevalence, contradicting a previous report by Hibbeln and colleagues (31). Specifically, the previous study correlated DHA in the milk of breastfeeding mothers to PPD rates by country (*N* = 23), and found that countries that consumed more seafood tended to have lower rates of PPD prevalence (31). There are several possible reasons for the discrepancy between our current findings and this previous report. For example, the current study examines twice the number of countries (*N* = 46) and includes countries with more diverse economic conditions. Additionally, the current study used meta-regression which weighted countries and studies, and controlled for methodological variables and GDP, while Hibbeln (31) did not control for these confounding variables. Although our findings were not significant, the effect we found was in the same direction as Hibbeln (31). Additionally, Hibbeln (31) was published over two decades ago, and it is possible that breastfeeding women were not discouraged from consuming certain seafood in the same way they are now due to concerns surrounding mercury poisoning (165). Future directions focusing on PPD and seafood consumption, particularly seafood high in omega-3 fatty acids, is warranted. In addition to seafood consumption, we found no relationships between fiber, yogurt, fruit, and vegetable consumption and national PPD prevalence. Despite these null results at the cross-national level, we encourage researchers to continue to examine the relationship between these dietary factors and PPD.

### 4.1. Implications

Our results have several important implications for research and policy to prevent PPD. For example, our research suggests that policies that help reduce SSB consumption may help reduce PPD prevalence. There are several existing policies shown to reduce SSB consumption at the national and regional level. For example, implementing a “sugar tax” at the national level disincentivizes the consumer from purchasing SSB by increasing the overall price. Therefore, causing a loss in SSB revenues and encouraging SSB companies to reduce the sugar in their product so that it does not qualify as a high-sugar product. In addition, the extra tax revenue from this strategy can then be used to offset the health care costs associated with sugar-related disease (166). Currently, 45 countries in the world have some type of sugar tax, however, in the US, sugar taxes are in place in only a few cities. Due to the availability of cross-national and within-country sugar consumption data, future research could explore whether implementing sugar taxes changes national rates of depression. While a sugar tax is a good start, more aggressive policies and behavioral health campaigns focusing on limiting SSB consumption may also be necessary. For example, interventions could focus on limiting the consumption of common ingredients in most SSBs. In the last 50 years, the use of high fructose corn syrup has increased dramatically, largely due to the fact that it is inexpensive and manufacturing friendly (167). National and local policies to reduce the use of high fructose corn syrup may be a viable step to improve maternal mental health. However, all types of added sugar are low in nutrient density, and high fructose corn syrup is just one factor in a massive global issue. Therefore, while focusing on types of added sugars in policymaking may lead to some health improvement, reducing consumption of all sugar types would be most impactful.

In addition to gaining a deeper understanding of the impact caused by different types of sweeteners, future work on this topic should focus on identifying additional cultural factors that may further explain variance in national PPD prevalence. For example, environmental and lifestyle factors such as exercise, obesity, partner support, and mindfulness practices, which have been linked to PPD, in individual studies might also be linked to PPD on the national level (168–171). Future research could also focus on micronutrients, such as vitamin D, that previous research have shown play an important role in mental health (172).

### 4.2. Limitations

Although this study had several strengths, including the large number of studies representing populations with varied lifestyles, economic backgrounds, and diets, our results should be considered alongside several important limitations. First, our study focused on a widely used self-report PPD measure instead of clinical interviews. Self-report PPD measures tend to yield higher estimates than clinical interviews, and therefore, the estimates in this study are likely higher than if we had used studies that conducted interview-based methods. However, given that interview methods are less common than self-report measures, especially in low-income countries, we felt that it was important to utilize a more representative measure. We also felt that potentially overestimating PPD would be safer than underestimating PPD, given its detrimental consequences. We use is nationally representative, and some countries estimates are much more reliable than others. We urge readers to consider the 95% confidence intervals when considering our national PPD prevalence estimates. More research reporting PPD prevalence is essential for providing more accurate prevalence estimates that. Secondly, collecting accurate nutrition data is notoriously challenging. This is why we used the Global Dietary Database, which has the most comprehensive empirical data on dietary intake across and within nations (173). Even so, it is difficult to identify inaccuracies within it. Thirdly, when using aggregate data, it is important to remember the ecological fallacy- that analyses based on area-level averages can yield very different conclusions than those that would be obtained from an analysis at the individual level (174).

## 5. Conclusion

PPD is common globally, however, there is significant cross-national and within-country variation. While certain methodological variables may contribute to this variation, SSB consumption may also be a risk factor. Future research is warranted to test whether policy that reduces SSB consumption at the national level may help to reduce PPD prevalence.

## Data availability statement

The raw data supporting the conclusions of this article will be made available by the authors, without undue reservation.

## Author contributions

AF-W conducted the review, meta analysis, and meta regression, as well as wrote the manuscript. JH-H oversaw all analysis, edited the manuscript, and provided guidance. All authors contributed to the article and approved the submitted version.

## Supplementary material

Figure S1 Forest plot of between-study variation in PPD prevalence.

## Appendix

Search Syntax

CINAHL 7/26/21 Here is the Boolean search that yields 195

records: Boolean search

(IN edinburgh postnatal depression scale) AND (AB (postpartum depression OR postnatal depression)) AND (AB (incidence OR prevalence))

With the additional limiters:

(1) Narrow to start 2016
(2) Narrow it to English articles only
(3) Narrow it to Humans only
(4) Narrow it to females only

PSYCHINFO 7/26/21 Here is the Boolean search that yields 162

records: AB (incidence or prevalence) AND AB (postnatal depression or postpartum depression) AND

TM edinburgh postnatal depression scale Here are the limiters: Date: start 2016

* Language: English
* Population: Female

PUBMED 7/26/21

Here is the Boolean search that yields 245 records: ((“postpartum depression”[All Fields] OR “postnatal depression”[All Fields]) AND “prevalence”[All Fields]) AND “edinburgh postnatal depression scale”[All Fields] AND ((“2015/01/01”[PDAT]) AND “humans”[MeSH Terms])
