# Supplementary figures and images for: Nutritional factors and cross-national postpartum depression prevalence: an updated meta-analysis and meta-regression of 412 studies from 46 countries
Source: Front Psychiatry. 2023 Jun 15;14:1193490. doi: 10.3389/fpsyt.2023.1193490 (PMC10311512; doi:10.3389/fpsyt.2023.1193490)

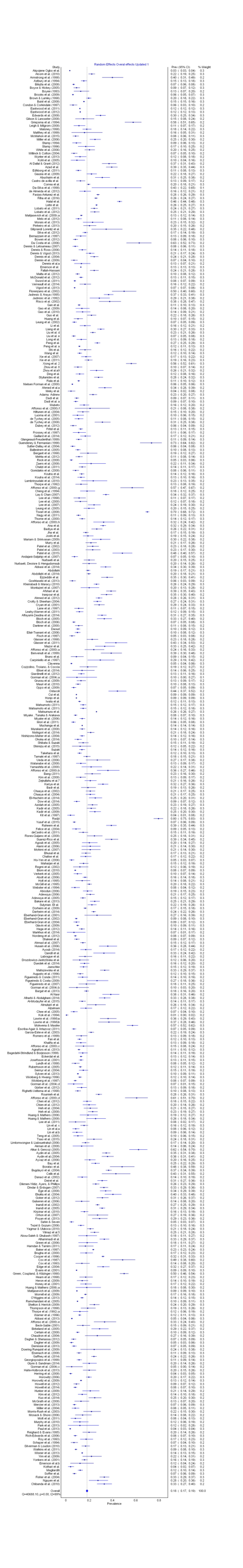

Supplement: Figure S1 — Forest plot of between-study variation in PPD prevalence. [file Image_1.jpeg]
